# Downregulation of CYP2A6 and CYP2C8 in Tumor Tissues Is Linked to Worse Overall Survival and Recurrence-Free Survival from Hepatocellular Carcinoma

**DOI:** 10.1155/2018/5859415

**Published:** 2018-07-25

**Authors:** Xiaojing Ren, Yuanyuan Ji, Xuhua Jiang, Xun Qi

**Affiliations:** Department of Liver Disease, Shanghai Public Health Clinical Center, Fudan University, Shanghai 201508, China

## Abstract

**Objective:**

This study aimed to evaluate the links between CYP450 family genes in tumor tissues and hepatocellular carcinoma (HCC) outcomes.

**Methods:**

Gene Expression Omnibus (GEO) databases GSE14520 and GSE36376 were used to identify differential expressed CYP450 genes between tumor and nontumor tissues and related to HCC clinicopathological features and survivals.

**Results:**

Seven CYP450 genes including CYP1A2, CYP2A6, CYP2C8, CYP2C9, CYP2E1, CYP3A4, and CYP4A11 were downregulated in tumor tissues, which were validated in both GSE14520 and GSE36376. HCC patients with CYP2A6 and CYP2C8 low levels in tumor tissues suffered from poorer overall survival (OS) compared to those with high CYP2A6 and CYP2C8 in GSE14520 profile (log ranks* P* = 0.01 and* P* = 0.006, respectively). In addition, HCC patients with lower CYP2A6 and CYP2C8 in tumors had worse recurrence-free survival (RFS) than those with higher CYP2A6 and CYP2C8 (log ranks* P* = 0.02 and* P* = 0.012, respectively). In GSE36376 validation dataset, HCC patients with lower CYP2A6 and CYP2C8 had worse OS and RFS than those with higher CYP2A6 and CYP2C8 (all* P* < 0.05), in line with results in GSE14520 dataset. Additionally, lower CYP2A6 and CYP2C8 are associated with advanced clinicopathological features including tumor staging, vascular invasion, intrahepatic metastasis, and high alpha fetoprotein (all* P* < 0.05).

**Conclusion:**

Downregulation of CYP2A6 and CYP2C8 in tumor tissues links to poorer OS and RFS in HCC patients.

## 1. Introduction

Accounting for 85-90% of primary liver malignancy, hepatocellular carcinoma (HCC) is the fifth most common tumor worldwide and the second most common cause of cancer-related death [1–4]. Over the last 20 years, the incidence of HCC has been rising in the United States and China [1, 2]. Given the complexity of the disease and the large number of useful therapies, patients with HCC still suffered from poor prognosis [5]. To find novel biomarkers for predicting outcomes and efficacy of anticancer agents is of great essential.

The efficacy of anticancer therapy is limited by our inability to predict patient outcomes such as tumor response and toxicity. Drug-metabolizing enzymes in tumor tissues play critical roles in the activation or inactivation of numerous cytotoxic drugs and influence the susceptibility of host and neoplasm to their effects [6]. Cytochrome P450s (CYPs) is a large group of enzymes that localize to mitochondrial membranes or the endoplasmic reticulum and play crucial roles in the metabolism of endogenous and exogenous molecules, including most drugs [7]. It has been suggested that the local expression of CYPs in tumors is very important for the management of cancer since these functionally associated enzymes might be involved both in the development of HCC and in determining the anticancer drug sensitivity of such tumor [8, 9].

Given the key roles of CYPs in tumor development, few data were available of CYPs in tumor tissues and HCC clinicopathological features and survivals. This study aimed to evaluate differential expressed CYP genes and to present predict roles of CYPs in tumor tissues for HCC survivals.

## 2. Materials and Methods

### 2.1. Patients

HCC patients in this study were from cohorts in GSE14520 and GSE36376. In GSE14520, 247 HCC patients were identified. Data on gene expression could not be obtained for 22 of these and a further 5 had insufficient clinical outcome data available, leading to 220 patients being included in the analysis. Cases consisted of patients with a history of hepatitis B virus (HBV) infection or HBV-related liver cirrhosis; the diagnosis of HCC was made in all cases by two independent pathologists who had detailed information on clinical presentation and pathological characteristics [10]. In GSE36376, 240 tumor tissues containing no necrosis or hemorrhage were available from primary HCC patients who were treated with surgical resection or liver transplantation at Samsung Medical Center, Seoul, Korea, from July 2000 to May 2006. None of the patients received preoperative chemotherapy.

### 2.2. Ethics Statement

In GSE14520, all liver tissue was obtained with informed consent from patients who underwent radical resection between 2002 and 2003 at the Liver Cancer Institute and Zhongshan Hospital (Fudan University). The study was approved by the Institutional Review Board of the participating institutes [10]. All participants provided written informed consent, as reported by Roessler et al. [10, 11]. In GSE36376, informed consent was obtained from each patient, and the study protocol was approved by the Institutional Review Board of Samsung Medical Center, Seoul, Korea, which is in agreement with reports by Lim HY et al. [12]. The second analysis protocol was approved by the Ethics Committee of Shanghai Public Health Clinical Center, Fudan University.

### 2.3. Source of Data

We extracted the GSE14520 and GSE36376 microarray expression profiles from Gene Expression Omnibus (GEO, http://www.ncbi.nlm.nih.gov/geo/) database. For GSE14520, tumor sample and microarray processing were reported by Roessler et al. [10, 11]. Gene expression levels were calculated using the matchprobes package in the R programming environment and the log2 RMA-calculated signal intensity was reported. Details of the experiment protocols and data processing are available at http://www.ncbi.nlm.nih.gov/geo/query/acc.cgi?acc=GSM362949. For GSE36376, tumor tissues of HCC patients after curative hepatectomy were profiled using Illumina HumanHT-12 V4.0 expression beadchip (Illumina Inc., San Diego, CA). The expression data was retrieved from Gene Expression Omnibus (GSE14520 and GSE36376, http://www.ncbi.nlm.nih.gov/geo/) [10–12]. We used GEO2R tool for identifying differential expressed genes with criteria of |log FC| > 2 and adjust* P* < 0.0001.

### 2.4. Definitions of Outcomes

Overall survival (OS) was defined as the time from surgery to death from any disease. Recurrence-free survival (RFS) was defined as time from surgery to the date of tumor recurrence or death. In GSE14520, RFS was diagnosed by new lesions found in the computed tomography (CT) or magnetic resonance imaging (MRI) scans and an abnormal alpha-fetoprotein (AFP) value with a cut-off of 300 ng/ml, including instances when the high pretreatment AFP value did not decrease to a normal level or increased again after becoming normal. In GSE36376, patient serum AFP levels were evaluated and three-phase dynamic CT scans were performed at least once every 3 months after surgery until December 31, 2010. When tumor recurrence was suspected, precise diagnostic imaging was performed by MRI.

### 2.5. Statistical Analysis

Student's* t*-test was used to compare means for normally distributed continuous data, and the Mann–Whitney* U* test was used for nonnormally distributed continuous data and the Chi-squared test was used for categorical variables. The Kaplan–Meier method was used to compare survivals between different groups, and the log-rank test was used to estimate the difference in survival. Statistical analyses were performed using PASW Statistics software version 23.0 from SPSS Inc. (Chicago, IL, USA). A two-tailed* P* < 0.05 was considered statistically significant.

## 3. Results

### 3.1. Differential Expressed CYPs in Both GSE14520 and GSE36376

**Figure 1:**
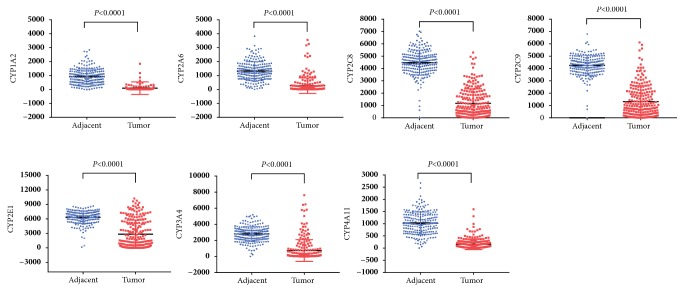
Differential expressed CYPs in GSE14520 profile.

**Figure 2:**
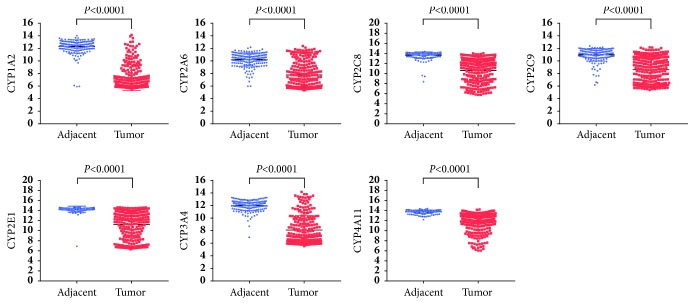
Differential expressed CYPs in GSE36376 profile.

As shown in Table 1, eleven CYPs including CYP1A2, CYP2A6, CYP2B6, CYP2B7P, CYP2C8, CYP2C9, CYP2E1, CYP3A4, CYP4A11, CYP4A22, and CYP39A1 were identified differential expression in GSE14520 dataset. In GSE36376, eight CYPs including CYP1A2, CYP2A6, CYP2C8, CYP2C9, CYP2E1, CYP3A4, CYP4A11, and CYP8B1 were significantly differentially expressed. Combinedly, seven CYPs including CYP1A2, CYP2A6, CYP2C8, CYP2C9, CYP2E1, CYP3A4, and CYP4A11 were all differentially expressed in both GSE14520 and GSE36376 datasets. As shown in Figures 1 and 2, all the seven differential CYPs were lower expressed in tumor tissues compared to those in nontumor tissues.

### 3.2. CYP Genes Associated with OS in HCC

**Figure 3:**
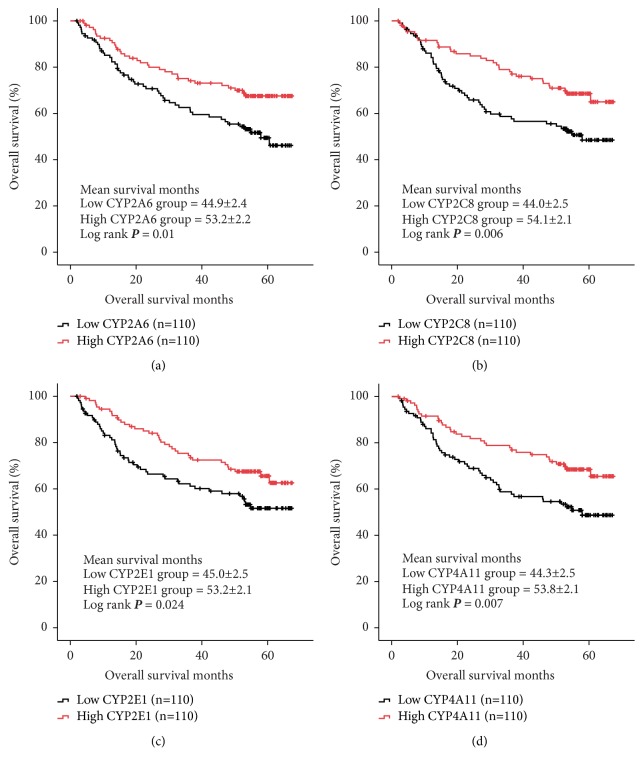
Overall survival (OS) comparison grouped by CYP2A6 (a), CYP2C8 (b), CYP2E1 (c), and CYP4A11 (d) from GSE14520.

**Figure 4:**
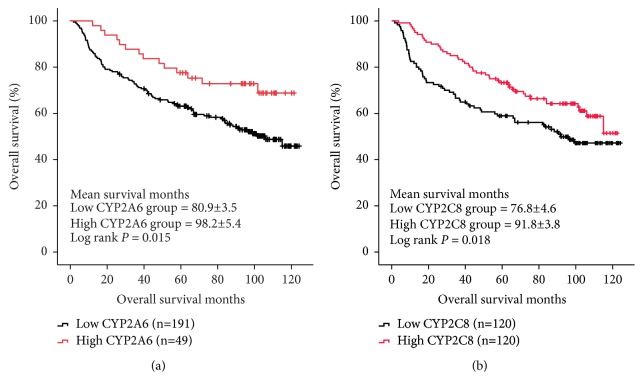
Validation of CYP2A6 (a) and CYP2C8 (b) for OS in database GSE36376.

In GSE14520 dataset, we identified the fact that low expressions of four CYPs including CYP2A6, CYP2C8, CYP2E1, and CYP4A11 in tumor tissues were significantly associated with worse OS in HCC patients (log ranks* P* = 0.01, 0.006, 0.024, and 0.007, respectively, Figure 3). Furtherly, we conducted Kaplan–Meier curve for validation of the four CYPs (CYP2A6, CYP2C8, CYP2E1, and CYP4A11) for evaluating its links to OS in HCC patients from GSE36376 dataset. As shown in Figure 4, downregulation of CYP2A6 was significantly associated with OS in HCC patients with cut-off of 10.0 detected by R program (log rank* P* = 0.015, Figure 4(a)). And, low expression of CYP2C8 in tumor tissues was linked to worse OS in HCC patients with median cut-off (log rank* P* = 0.018, Figure 4(b)).

### 3.3. CYP Genes Associated with RFS in HCC

**Figure 5:**
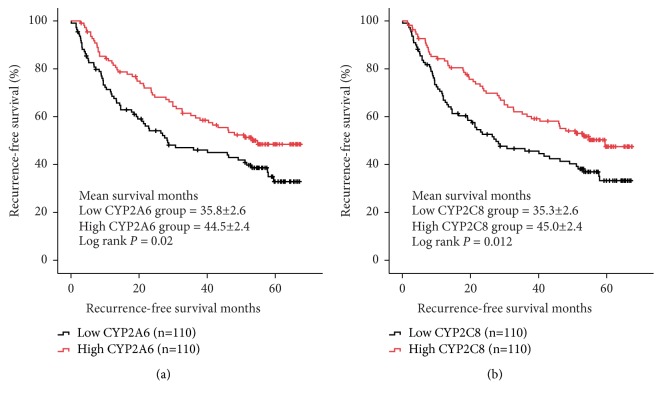
Recurrence-free survival (RFS) comparison grouped by CYP2A6 (a) and CYP2C8 (b).

**Figure 6:**
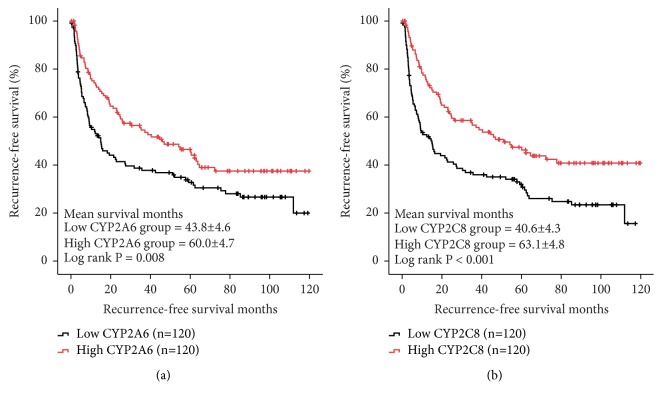
Validation of CYP2A6 (a) and CYP2C8 (b) for RFS in database GSE36376.

In GSE14520 dataset, low expressions of two CYPs including CYP2A6 and CYP2C8 were significantly associated with worse RFS in HCC patients (log ranks* P* = 0.02 and 0.012, respectively, Figure 5). In validation set from GSE36376, downregulation of CYP2A6 and CYP2C8 was also related to poorer RFS in HCC patients (log ranks* P* = 0.008 and* P* < 0.001, respectively, Figure 6).

### 3.4. Relationship between CYP2A6, CYP2C8, and HCC Clinicopathological Features

In GSE14520 profile, HCC patients had advanced TNM staging in CYP2A6 and CYP2C8 low expression groups compared to those in CYP2A6 and CYP2C8 high expression groups (*P* = 0.005 and* P* = 0.032, respectively, Table 2). Interestingly, in GSE36376 database, HCC patients with low CYP2A6 and CYP2C8 levels in tumor tissues suffered from more advanced edmondson grade and AJCC staging (all* P* < 0.05, Table 2). Additionally, HCC patients with low CYP2A6 and CYP2C8 levels in tumor tissues had higher risk of vascular invasion, major portal vein invasion, and intrahepatic metastasis (all* P* < 0.05, Table 2). Also, those HCC cases with low CYP2A6 and CYP2C8 had significantly higher AFP level (*P* < 0.001, Table 2). Considered above, we assumed that downregulation of CYP2A6 and CYP2C8 in tumor tissues is associated with worse clinicopathological characteristics in HCC patients.

## 4. Discussion

As key enzymes in cancer formation and cancer treatment, CYPs mediate the metabolic activation of multiple procarcinogens and participate in the inactivation and activation of anticancer drugs [13]. CYPs polymorphisms have been extensively studied with respect to genetic predisposition to cancer and clinical outcome in terms of response and toxicity to anticancer drugs [14]. Various studies have also shown the significance of CYPs polymorphisms in cancer susceptibility [15–18]. Some members of CYPs family also affect the metabolism of various environmental carcinogens [14]. In HCC patients, CYP1A1, CYP2D6, and CYP2E1 were all found to be associated with increased HCC risk in different population [19–21]. However, a consistent view does not yet exist [14, 22].

CYPs are key enzymes involved in cancer development and mediate the metabolic activation of numerous procarcinogens [13]. To assess the CYPs activity changes would be useful not only for designing personalized HCC treatments, but also for identifying potential factors that contribute to HCC susceptibility [23]. In our study, we found that several CYPs including CYP1A2, CYP2A6, CYP2C8, CYP2C9, CYP2E1, CYP3A4, and CYP4A11 were all downregulated in tumor tissues in HCC patients. And, low expression of CYP2A6 in tumor tissues contributed to worse survivals from HCC cases. CYP2A6 is primarily expressed in the liver and metabolizes several clinically relevant substrates [24]. Variation in CYP2A6 activity is involved in the metabolism or bioactivation of clinical therapeutics and carcinogens, leading to an important clinical consideration [25–27]. CYP2A6 was also associated with worse clinicopathological characteristics including advanced tumor staging, vascular invasion, major portal vein invasion, intrahepatic metastasis, and increased AFP level in our study. In line with our findings, CYP2A6 is highly polymorphic and its genetic variants can result in reduced expression by affecting transcriptional or translational processes [28]. Clinically, genetic variation in CYP2A6 may contribute to lung cancer risk [29, 30]. However, few studies focused on the relationships between CYP2A6 and HCC development. Raunio H et al. investigated the distribution of the CYP2A6 protein in a series of 24 human hepatocellular carcinoma (HCC) samples by immunohistochemical analysis. They found that CYP2A6 protein was very heterogeneous in tumor cells and HCC do not uniformly overexpress the CYP2A6 protein, suggesting that increased expression of CYP2A6 occurred in a distinct subpopulation of neoplastic cells. Additionally, they found that patients with CYP2A6-positive tumors achieved a more favorable prognosis compared with patients with CYP2A6-negative tumors [31]. Our results conformed and replenished the previous research [8, 31]. More detailed studies on the association between CYP2A6 and HCC should be conducted.

Several genetic polymorphisms in CYP2C8 may influence survival after cancer diagnosis due to their role in the metabolism of various breast cancer chemotherapy drugs [32]. However, the risk of developing colorectal cancer does not seem to be related to the commonest functional genetic variation in the CYP2C8 gene [33]. Unfortunately, knowledge on CYP2C8 in tumors and HCC outcomes is limited. Our research demonstrated that CYP2C8 downregulation in tumor tissues was associated with advanced tumor phenotype and worse OS and RFS. Yan et al. determined seven CYPs including CYP2C8 in tumor and pericarcinomatous tissues harvested from 26 patients with HBV-positive HCC using probe substrates. A major decrease of CYP2C8 in tumor tissues was observed [6], in line with a study by Zhou et al. [23]. Experimentally, polycyclic aromatic hydrocarbons 3-methylcholanthrene suppressed CYP2C8 mRNA levels in the HCC cell line HepG2, and basal CYP2C8 expression was extremely low [34]. That is, CYP2C8 expression levels are greatly disrupted by the tumorigenic process [6]. Since drug metabolism mediated by tumor CYPs can be used as a marker for potential mechanism of drug resistance and/or an approach to achieve optimal chemotherapy, to upregulate CYP2C8 levels, and activations should be considered for HCC treatment strategy.

There are some limitations in this study. Firstly, CYPs expression in our research is limited in mRNA level, which may not necessarily reflect that in activities of CYPs. Secondly, in our analysis based on GEO database, no further mechanism data were shown.

According to our results, we cautiously drew the conclusion that downregulation of CYP2A6 and CYP2C8 in tumor tissues is linked to worse overall survival and recurrence-free survival from hepatocellular carcinoma. A detailed understanding of the differential expression and activity of CYPs within HCC tumor tissues may provide opportunities and more alternatives for improved therapeutic outcome.

## Figures and Tables

**Table 1:** Differential expressed CYP450 genes between tumor and nontumor tissues from GSE14520 and GSE36376.

Genes	t	B	log⁡FC	*P* value	Adjust *P* value	Common genes
**GSE14520**						CYP1A2 CYP2A6 CYP2C8 CYP2C9 CYP2E1 CYP3A4 CYP4A11
CYP1A2	25.7352762	49.901907	4.44705742	1.63E-22	7.31E-27	
CYP2A6	7.9627779	12.348032	3.07188278	3.66E-08	7.66E-10	
CYP2B6	15.7936233	33.005014	3.89306938	6.65E-16	5.37E-19	
CYP2B7P	24.284837	180.032064	3.5900407	1.8E-81	1.25E-83	
CYP2C8	6.4917335	7.665054	4.32698325	0.0000018	8.78E-08	
CYP2C9	23.3715192	46.568366	3.36577273	1.63E-21	2.93E-25	
CYP2E1	8.2816633	13.340866	4.73015789	0.000000016	2.81E-10	
CYP3A4	7.9970664	12.45527	3.99461005	3.37E-08	6.87E-10	
CYP4A11	8.8192119	14.989899	3.11363397	3.99E-09	5.29E-11	
CYP4A22	8.6445676	14.457718	3.17968421	6.1E-09	9.06E-11	
CYP39A1	10.5703094	20.114355	3.19955263	5.84E-11	2.94E-13	
**GSE36376**						
CYP1A2	30.5006424	240.408848	4.97	1.29E-106	6.05E-110	
CYP2A6	15.0803249	83.643956	2.47	2.71E-40	1.3E-41	
CYP2C8	17.410711	107.141675	3.05	2.5E-50	7.36E-52	
CYP2C9	14.9361339	82.220342	2.36	1.1E-39	5.44E-41	
CYP2E1	15.0716592	83.558278	3	2.95E-40	1.42E-41	
CYP3A4	22.4720068	159.580681	3.91	1.3E-72	1.03E-74	
CYP4A11	14.3069129	76.061125	2.06	4.62E-37	2.65E-38	
CYP8B1	12.9803487	63.40673	2.18	1.18E-31	8.9E-33	

**Table 2:** Clinicopathological comparison grouped by CYP2A6 and CYP2C8 from GSE14520 and GSE36376.

Category	CYP2A6		*P* value	CYP2C8		*P* value
	High	Low		High	Low	
**GSE14520**						
HBV viral status (AVR-CC/CC/NA)	25/81/4	31/74/5	0.635	28/78/4	28/77/5	0.943
Alanine aminotransferase (>50/<50, U/L)	38/72	51/59	0.099	42/68	47/63	0.583
Main tumor size (>5/<5/NA, cm)	34/76/0	44/65/1	0.16	33/77/0	45/64/1	0.091
Multinodular (yes/no)	18/92	27/83	0.181	21/89	24/86	0.738
Cirrhosis (yes/no)	101/9	101/9	1.0	101/9	101/9	1.0
TNM staging (I/II/III/NA)	57/37/15/1	36/40/33/1	**0.005**	56/33/21/0	37/44/27/2	**0.032**
BCLC staging (0-A/B/C/NA)	83/9/7/1	75/13/21/1	*0.039*	79/8/13/0	79/14/15/2	0.357

**GSE36376**						
Edmondson grade (I/II/III)	16/100/4	8/97/15	**0.01**	19/96/5	5/101/14	**0.002**
Tumor size (median), mm	34 (31)	40 (45)	0.203	34 (30)	42 (45)	0.081
Vascular invasion (yes/no)	51/69	84/36	**<0.001**	51/69	84/36	**<0.001**
Major portal vein invasion (yes/no)	1/119	8/112	**0.036**	1/119	8/112	**0.036**
Intrahepatic metastasis (yes/no)	17/103	38/82	**0.002**	18/102	37/83	**0.005**
Multicentric occurrence (yes/no)	8/112	5/115	0.57	7/113	6/114	1.0
Direct invasion of adjacent organ (yes/no)	3/117	2/118	1.0	3/117	2/118	1.0
AJCC staging (1/2/3/4)	66/43/8/3	36/57/25/2	**<0.001**	67/42/8/3	35/58/25/2	**<0.001**
BCLC staging (A/B/C)	75/43/2	64/48/8	0.092	80/38/2	59/53/8	*0.009*
Alpha fetoprotein [Median (IQR), ng/ml]	9.65 (82.2)	256.6 (4136.8)	**<0.001**	9.1 (61.6)	303.8 (9534.45)	**<0.001**

NA: not available; AVR-CC: active viral replication chronic carrier; CC: chronic carrier; IQR: interquartile range;

## Data Availability

All the data in this study were available from GEO database (https://www.ncbi.nlm.nih.gov/geo/).
